# Supplementary material for: Intermolecular Vibration Energy Transfer Process in Two CL-20-Based Cocrystals Theoretically Revealed by Two-Dimensional Infrared Spectra
Source: Molecules. 2022 Mar 26;27(7):2153. doi: 10.3390/molecules27072153 (PMC9000797; doi:10.3390/molecules27072153)
Supplement: Supplementary file 1 [file molecules-27-02153-s001.zip › molecules-1632610-supplementary.pdf]

Supporting Information

Intermolecular Vibration Energy Transfer Process in Two CL-20-Based Cocrystals Theoretically Revealed by Two-Dimensional Infrared Spectra

Hai-Chao Ren[1], Lin-Xiang Ji[2], Tu-Nan Chen[3], Xian-Zhen Jia[1], Rui-Peng Liu[1], Xiu-Qing Zhang[4], Dong-Qing Wei[5, 6], Xiao-Feng Wang[1, *] and Guang-Fu Ji[7,*]

[1]Xi'an Modern Chemistry Research Institute, Xi'an 710065, China

[2]Department of Physics and Engineering Physics, University of Saskatchewan, Saskatoon, Saskatchewan, S7N5E2, Canada

[3]The Southwest hospital of AMU, Army Medical University, Chongqing 400038, China

[4]School of Science, North University of China, Taiyuan 030051, China

[5]College of Food Science and Engineering, Henan University of Technology, Zhengzhou, 450001, China

[6]College of Life Science and Biotechnology, Shanghai Jiao Tong University, Shanghai 200240, China

[7]National Key Laboratory for Shock Wave and Detonation Physics Research, Institute of Fluid Physics, Chinese Academy of Engineering Physics, Mianyang 621999, China

[*] Corresponding Author Emails: wxfclub@163.com; cyfjkf@caep.cn

Table S1. Coordinates (Å) of TNT/CL-20 and HMX/CL-20 shown in Figure 1

TNT/CL-20				HMX/CL-20			
Element	X	Y	Z	Element	X	Y	Z
O	4.587097	0.59393	2.30159	O	5.840077	-2.578102	-2.500406
O	3.776864	1.467461	0.490611	O	3.891175	1.341431	-2.919977
O	0.148842	4.474325	1.022817	O	2.189333	0.06136	-3.380088
O	-1.509275	3.915019	2.306981	O	0.176057	-1.304159	0.580426
O	-1.233347	-0.483511	4.475075	O	0.995089	0.011442	2.115295
O	0.571015	-0.538219	5.687876	O	3.184173	-4.345343	2.410487
O	-1.219696	-3.199216	-3.94579	O	2.170018	-4.868373	0.559981
O	-0.132855	-4.689388	-2.790017	O	4.225624	-1.368822	3.878026
O	1.482989	-0.895464	0.675294	O	3.66059	0.687405	3.435065
O	0.487059	0.613699	-0.529857	O	7.582574	-1.096376	-0.463814
O	-2.641255	-5.75324	-1.265216	O	6.801225	0.830444	-1.111135
O	-4.248083	-5.115816	0.073733	N	4.283056	-2.764038	-0.930647
O	-4.535903	-1.9013	-3.158473	N	4.660645	-2.763492	-2.275756
O	-5.757478	-1.975602	-1.359398	N	3.420721	-0.377823	-1.562168
O	0.005953	-2.832786	3.146007	N	3.124332	0.430121	-2.713417
O	-2.044052	-3.373756	3.63411	N	2.104395	-0.314614	0.236725
O	-1.947228	0.960373	1.130712	N	1.039776	-0.5533	1.039883
O	-3.537041	-0.168889	2.11654	N	2.915028	-2.743279	0.844256
N	3.68462	1.104609	1.654663	N	2.755095	-4.118566	1.30169
N	-0.381856	3.759406	1.862991	N	3.929381	-0.755081	1.749106
N	-0.069338	-0.191435	4.71217	N	3.923002	-0.453027	3.141342
N	-0.862802	-3.735988	-2.919512	N	5.398478	-0.685964	-0.272363
N	-1.34241	-3.149671	-1.722545	N	6.699196	-0.291493	-0.662552
N	0.546671	-0.47097	0.011979	C	5.118697	-2.060809	0.055807
N	-0.517784	-1.341143	-0.164071	C	4.167629	-2.099376	1.316319
N	-3.113389	-5.089572	-0.366385	C	2.866111	-2.586752	-0.600095
N	-2.23636	-4.207259	0.274584	C	2.343042	-1.126617	-0.962227
N	-4.719109	-2.050457	-1.972184	C	4.272482	0.178859	-0.504912
N	-3.557023	-2.443055	-1.201843	C	3.375081	0.169563	0.796188
N	-1.176682	-2.97011	2.903164	H	6.059405	-2.57428	0.216664
N	-1.589241	-2.522939	1.59847	H	4.57514	-2.670871	2.14228
N	-2.772985	0.058984	1.205495	H	2.243825	-3.342701	-1.062379
N	-2.858772	-0.765515	0.097173	H	1.474226	-1.165226	-1.608603
C	2.657011	-0.812439	3.72311	H	4.606387	1.173441	-0.775206
H	1.985282	-1.571718	4.109786	H	3.270391	1.149731	1.247068
H	3.36065	-0.543632	4.514048	O	-0.645218	-3.974666	0.444397
H	3.241465	-1.228803	2.906801	O	-2.2302	6.595661	0.22872
C	1.899622	0.405525	3.279332	O	-1.003901	6.521508	2.032252
C	2.38059	1.311779	2.31545	O	-1.297916	3.484168	-3.555072

C	1.663015	2.401392	1.852306	O	-2.766862	2.607291	-2.204132
H	2.068599	3.041189	1.084514	N	-1.056282	-3.351333	-0.50398
C	0.399781	2.621367	2.366522	N	-1.436265	4.635572	0.927227
C	-0.165984	1.758317	3.287551	N	-1.577457	6.017371	1.080743
H	-1.181052	1.896728	3.627333	N	-1.332051	4.071758	-1.398529
C	0.598925	0.690406	3.728387	N	-1.816177	3.337486	-2.467498
C	-1.04364	-3.724827	-0.444363	C	-2.131467	3.996853	-0.160455
H	-0.299831	-4.500895	-0.578153	C	-0.714029	3.910536	1.942079
C	-0.560421	-2.594206	0.544419	H	-2.281067	2.958902	0.112297
H	0.410588	-2.805234	0.972529	H	-3.096002	4.475287	-0.318141
C	-1.725191	-0.901336	-0.819868	H	-1.080273	2.88854	1.951682
H	-1.511776	0.003237	-1.372091	H	-0.885974	4.364709	2.915327
C	-2.247165	-2.047292	-1.767746	O	2.436791	2.598272	-0.423123
H	-2.363608	-1.713385	-2.793248	O	1.161375	2.583542	-2.187687
C	-2.806296	-3.118317	1.071437	O	0.939963	2.754787	3.495033
H	-3.43885	-3.491792	1.866202	O	2.747384	3.39701	2.45342
C	-3.578906	-2.038795	0.191874	N	1.016038	4.270133	-0.749163
H	-4.590987	-1.896129	0.548356	N	1.568483	3.070046	-1.152581
O	1.42486	-1.943749	-2.38766	N	0.730158	3.910985	1.633154
O	2.102302	-3.418624	-0.963061	N	1.536495	3.317849	2.590494
O	5.5833	-2.436458	2.015934	C	1.33505	4.721947	0.596692
O	7.237912	-1.17914	1.380084	C	-0.09257	4.797511	-1.539481
O	6.155991	2.03733	-2.19903	H	1.004482	5.75761	0.667339
O	5.823796	0.860041	-3.995033	H	2.407183	4.676396	0.756427
N	2.266007	-2.397371	-1.622289	H	-0.222547	5.834876	-1.234619
N	6.137129	-1.678065	1.232452	H	0.165269	4.764977	-2.593749
N	5.745336	1.054951	-2.792001	O	3.769481	-2.979831	-3.075412
C	3.121996	-0.174862	-3.501065	O	-0.571917	-3.28099	-1.618874
H	3.667093	0.546167	-4.097188	O	-6.760565	-4.010799	-0.413352
H	2.852789	-1.018843	-4.136548	O	-6.009235	-3.634047	-2.419807
H	2.185672	0.269772	-3.161658	O	-4.665812	0.5249	-2.763037
C	3.931756	-0.630956	-2.31872	O	-5.298035	1.523127	-0.921318
C	3.530036	-1.657046	-1.435829	O	-0.895215	1.164845	-0.250076
C	4.245708	-2.023359	-0.302912	O	-0.859777	0.07655	-2.131157
H	3.888019	-2.809845	0.341548	O	-3.139866	1.757288	2.007973
C	5.403437	-1.339984	-0.003654	O	-5.29163	1.442598	2.059639
C	5.866899	-0.322749	-0.817053	O	-2.81014	-4.170534	2.755948
H	6.756063	0.231116	-0.556177	O	-4.941842	-4.385294	2.368241
C	5.146336	-0.019442	-1.959731	N	-2.287648	-2.673338	-0.304714
O	-0.945826	7.287199	-0.13185	N	-4.824571	-2.879915	-0.659018
O	-0.361017	7.101852	-2.214615	N	-5.978232	-3.548634	-1.212712
O	0.605077	2.484811	-3.383169	N	-5.031595	-0.67846	-0.945754
O	-1.004227	1.055483	-3.118471	N	-4.980762	0.560329	-1.593755
O	-4.580788	1.990312	-0.241017	N	-2.443949	-0.400403	-0.625229

O	-4.465627	3.620434	1.179332	N	-1.299411	0.319441	-1.033805
N	-0.887421	6.689054	-1.192072	N	-3.984744	-0.140481	1.194061
N	-0.481097	2.152795	-2.943706	N	-4.159868	1.123385	1.783204
N	-4.111481	3.04763	0.166818	N	-3.853901	-2.610519	1.532516
C	-3.370211	5.985786	0.313471	N	-3.865177	-3.838644	2.267507
H	-4.430568	5.751068	0.333263	C	-2.579119	-2.148865	1.03426
H	-3.00241	5.947382	1.34115	C	-2.667076	-0.581078	0.8191
H	-3.219123	6.99329	-0.061801	C	-2.714394	-1.684274	-1.27719
C	-2.61244	4.982658	-0.505146	C	-4.288257	-1.813565	-1.489368
C	-1.468194	5.32344	-1.252159	C	-4.998468	-2.330877	0.704825
C	-0.767297	4.437227	-2.052703	C	-5.097766	-0.767278	0.513003
H	0.109001	4.760772	-2.593544	H	-1.789128	-2.422223	1.722698
C	-1.219212	3.132216	-2.131074	H	-1.914432	-0.053241	1.390146
C	-2.339893	2.722729	-1.436841	H	-2.176176	-1.776445	-2.212597
H	-2.722184	1.721215	-1.516961	H	-4.520156	-1.971978	-2.534595
C	-2.992838	3.631485	-0.621606	H	-5.886551	-2.760091	1.153001
				H	-6.0194	-0.346931	0.89833

Table S2. Comparison of calculated (ω_{cal}, cm^{-1}) and experimental (ω_{exp}, cm^{-1}) infrared shifts[1-3] and vibrational mode assignments of TNT/CL-20.

$\omega_{cal.}$	$\omega_{exp.}$	Description
1534.9	1533	C-H bending in TNTIII and $N^{13}O_2$ as.
1588.6	1588	C-H bending in TNTI and TNTII, N^8O_2 and $N^{10}O_2$ as.
1596.3	1597	C-H bending in TNTII and TNTIII, $N^{12}O_2$ and $N^{13}O_2$ as.
1617.2	1619	C-H bending in TNTI and N^7O_2 as.
1630.4	1632	C-H bending in TNTII and $N^{11}O_2$ as.
1648.1		C-H bending in TNTII, N^8O_2 and $N^{10}O_2$ as.
1655.0		C-H bending in TNTIII, N^3O_2 and $N^{13}O_2$ as.
1666.2		C-H bending in TNTII and TNTIII, $N^{12}O_2$ and $N^{14}O_2$ as.
1669.8		N^2O_2 and N^3O_2 as.
1677.0		C-H bending in TNTI and N^9O_2 as.

. [a] Note: abbreviation used here is as follows: as = asymmetric stretching vibration

Table S3. Comparison of calculated (ω_{cal}, cm^{-1}) and experimental (ω_{exp}, cm^{-1}) infrared shifts[4-7] and vibrational mode assignments of HMX/CL-20

$\omega_{cal.}$	$\omega_{exp.}$	Description
1559.8	1563	N^1O_2, N^4O_2 and $N^{11}O_2$ as.
1606.3	1604	N^1O_2, N^4O_2 and $N^{11}O_2$ as.
1613.7	1615	N^1O_2, N^3O_2 and $N^{10}O_2$ as.
1643.5		N^5O_2, N^6O_2 and $N^{10}O_2$ as.
1659.2		N^1O_2, N^2O_2 and N^3O_2 as.
1661.0		N^7O_2, N^8O_2 and $N^{14}O_2$ as.
1676.8		N^8O_2, $N^{10}O_2$ and $N^{16}O_2$ as.
1681.6		N^5O_2 and N^6O_2 as.

[a] Note: abbreviation used here is as follows: as = asymmetric stretching vibration

Reference

1. Bolton, O.; Matzger, A. J. Improved stability and smart-material functionality realized in an energetic cocrystal. *Angew. Chem. Int. Edit.* **2011,** *123* (38), 9122-9125.

2. Yang, Z.; Zhang, Y.; Li, H.; Zhou, X.; Nie, F.; Li, J.; Huang, H. Preparation, structure and properties of CL-20/TNT cocrystal. *Chin. J. Energ. Mater.* **2012,** *20* (6), 674-679.

3. Hu, Y.; Yuan, S.; Li, X.; Liu, M.; Sun, F.; Yang, Y.; Hao, G.; Jiang, W. Preparation and characterization of nano-CL-20/TNT cocrystal explosives by mechanical ball-milling method. *Acs Omega* **2020,** *5* (28), 17761-17766.

4. Bolton, O.; Simke, L. R.; Pagoria, P. F.; Matzger, A. J. High power explosive with good sensitivity: a 2: 1 Cocrystal of CL-20: HMX. *Cryst. Growth Des.* **2012,** *12* (9), 4311-4314.

5. An, C.; Li, H.; Ye, B.; Wang, J. Nano-CL-20/HMX cocrystal explosive for significantly reduced mechanical sensitivity. *J. Nanomater.* **2017,** *2017*, Article ID 3791320, 7 pages.

6. Ghosh, M.; Sikder, A. K.; Banerjee, S.; Gonnade, R. G. Studies on CL-20/HMX (2: 1) cocrystal: a new preparation method and structural and thermo kinetic analysis. *Cryst. Growth Des.* **2018,** *18* (7), 3781-3793.

7. Hübner, J.; Deckert-Gaudig, T.; Glorian, J.; Deckert, V.; Spitzer, D. Surface characterization of nanoscale co-crystals enabled through tip enhanced Raman spectroscopy. *Nanoscale* **2020,** *12* (18), 10306-10319.
